# Reduction of breast tumor drug resistance by 2,3,5,4’-tetrahydroxystilbene for exhibition synergic chemotherapeutic effect

**DOI:** 10.1371/journal.pone.0260533

**Published:** 2021-12-07

**Authors:** Yao-Yuan Chang, Hung-Jun Lin, Ling-Chi Hsiao, Yu-Feng Lin, Chih-Sheng Chang, Der-Zen Liu

**Affiliations:** 1 Graduate Institute of Biomedical Materials and Tissue Engineering, College of Biomedical Engineering, Taipei Medical University, Taipei, Taiwan; 2 Division of Nephrology, Department of Internal Medicine, National Taiwan University Hospital, Taipei, Taiwan; 3 Department of Horticulture Science, National Chiayi University, Chiayi City, Taiwan; 4 Medical and Pharmaceutical Industry Technology and Development Center, New Taipei, Taiwan; Lobachevsky University, RUSSIAN FEDERATION

## Abstract

Chemotherapy drugs have limited efficacy in breast cancer due to multidrug resistance generated by cancer cells against anticancer drugs. In this study, we developed a novel derivative, 2, 3, 5, 4‘-tetrahydroxystilbene (TG1) by modifying 2, 3, 5, 4‘-tetrahydroxystilbene-2-O-beta-D-glucoside (THSG). In-vivo zebrafish embryo tests revealed that TG1 showed low toxicity. The equitoxic combination of DOX or DTX with TG1 in MCF-7/Adr reduced the IC_50_ of DOX or DTX, and the combination index (CI) showed strong synergistic effects in the 1:3 molar ratio of DTX: TG1 and 1:5 molar ratio of DOX: TG1. Moreover, fluorescence images confirmed the cellular uptake of DOX when combined with TG1 in MCF-7/Adr. Western blotting analysis indicated downregulation of p-glycoprotein (P-gp) after MCF-7/Adr treated with TG1. In conclusion, the combined therapy of DTX or DOX and TG1 increases drug efficacy via suppressing the p-glycoprotein efflux pump. These results suggest that TG1 may have potential use for breast cancer patients, especially those with multidrug resistance.

## Introduction

Breast cancer is one of the most common cancers in the world, and has the highest mortality rate among all cancers in women [1]. Previous reports have shown that approximately 12.3% of women in the US will be diagnosed with breast cancer during their lifetime, and it was predicted that it will reach up to 22 million new cases worldwide in a decade [2–4]. Thus, treating breast cancer efficiently is always an important issue for researchers. Surgery, chemotherapy and radiation therapy are the major treatments for breast cancer patients currently. Chemotherapy is useful for treating various stages of breast cancer, and is used preoperatively to shrink the tumor or postoperatively to eliminate residual tumor cells after surgical removal [5, 6]. Many chemotherapy drugs are used for breast cancer treatment, including doxorubicin (DOX), paclitaxel, docetaxel (DTX) and others. However, anti-tumor efficacy is often limited by the multidrug resistance (MDR), ultimately resulting in the failure of chemotherapy [7, 8]. Thus, MDR has become a significant problem for breast cancer treatment.

MDR has affected more than 80% of breast cancer patients after chemotherapy [9, 10]. Upregulation of ATP-binding cassette transporters (ABC transporters) on breast cancer cells is the major mechanism of MDR. ABC transporters, including p-glycoprotein (P-gp), multidrug resistance-associated protein (MRP) and breast cancer resistance protein (BCRP) are cell membrane efflux pumps, and their role is to modulate intracellular drug concentrations [9, 11, 12]. However, overexpression of ABC transporters pumps anticancer drugs out of cancer cells from intracellular to extracellular, resulting in losing the anti-cancer ability and failure of chemotherapy.

In order to overcome the MDR effect, a chemosensitizer must be developed to inhibit overexpression of ABC transporters. Different inhibitors, such as ribozymes, siRNA and antisense oligonucleotides have been validated previously to successfully reduce the expression of ABC transporters [13–15]. However, promising chemosensitizers are still lacking in clinical treatment because of previously identified undesirable drawbacks such as low potency and high toxicity [16, 17]. Recently, researchers are focusing on natural plants extraction and subsequent structural modification to develop chemosensitizers with versatile applications and low toxicities [18, 19]. 2,3,5,4’-tetrahydroxystilbene (TG1) is a compound that was modified from 2,3,5,4’-tetrahydroxystilbene-2-O-β-D-glucoside (THSG), which is extracted from Agave sisalana, Polygonum multiflorum and Fallopia japonica. The aim of this study was to demonstrate the synergistic effects of a novel chemosensitizer, TG1, combined with an anti-cancer drug to overcome multidrug resistance in breast cancer treatment.

## Materials and methods

### Production of TG1

THSG was extracted from *Polygonum multiflorum* as described in a previous study [20]. Briefly, *Polygonum multiflorum* leaves, roots and rhizomes were pulverized and soaked in 60% methanol for one day. After filtration, the residues were twice extracted with 60% methanol, and then concentrated with a rotary evaporator. The aqueous solution was chromatographed on a Diaion HP-20 column (30cm id×90cm) eluted with H_2_O, 50% MeOH and 100% MeOH. The 50% MeOH eluate was chromatographed over a RH-18 column (10cm id×60cm) eluted with 0.05% trifluoroacetic acid-CH_3_CN (82:18) to produce THSG. To form a THSG solution (2.06 g, 5.62 mmol) in EtOH (50 mL) was added 1.0 N HCl (70 mL), refluxed for 14 hours. The mixture was cooled to room temperature and EtOH was removed under reduced pressure. The residue was extracted with ether, and the organic layers were collected, dried over MgSO_4_, concentrated under reduced pressure, and purified by column chromatography (silica gel; DCM/MeOH = 16/1) to produce TG1 as a brown solid. After flash column purification, TG1 was dissolved in MeOH and recrystallized with CHCl_3_ to produce brown-green powder (400 mg, 32% yield). The compound structure of TG1 was examined its ^1^H and ^13^C using nuclear magnetic resonance (NMR), (Bruker Avance DRX 500MHz, Bruker Corp., Billerica, MA, USA).

### Cells

Two different types of human breast cancer cell lines were used. The first was MCF-7 cells, a model human breast cancer cell line, which was purchased from Bioresource Collection and Research Center (BCRC, Taiwan), and the second was MCF-7/Adr cells, a human breast cancer multidrug resistant (MDR) cell line, which was kindly provided by Professor Jun-Jen Liu (Taipei Medical University, Taiwan). In addition, L929 human fibroblast cell lines were purchased from BCRC, Taiwan, for *in-vitro* toxicity examination.

MCF-7 cells were cultured in Minimum Essential Medium (Gibco; Invitrogen, Waltham, MA, USA) containing L-Glutamine, 2.2g/L sodium bicarbonate, 0.1mM non-essential amino acids solution, 1.0 mM sodium pyruvate, 10% fetal bovine serum (FBS), and 1% penicillin/streptomycin (PS). MCF-7/Adr cells were cultured in Dulbecco’s modified Eagle’s medium (Gibco) containing 10% FBS and 1% PS. L929 cells were cultured in RPMI containing 10% FBS and 1% PS. All cell lines were maintained in a humidified atmosphere with 5% CO_2_ in a 37˚C incubator and the culture medium was replaced every 2–3 days.

### Cell viability examination

Cell viability was analyzed by Thiazolyl Blue Tetrazolium Bromide (MTT, Sigma- Aldrich, St. Louis, MO, USA). The cells were first placed in 48-well plates (Nunc) at a density of 1x10^4^ cells/well. After incubation for cells attached in the 48-well plates, each well would be placed into different drug conditions for 24 hours. Since the drugs were insoluble in water, they were dissolved in 0.1% dimethyl sulfoxide (DMSO, Sigma Aldrich, St. Louis, MO, USA). Then, each well would be substituted by the culture medium containing 10% MTT solution, which were made up in 5 mg/ml solution operated in dark place. After incubation for 4 hours, the supernatants were removed and 500 μl DMSO was added into each well to solubilize the formazan crystals. Finally, they were transferred into 96-well plates and then the absorption wavelength was measured at 570 nm by ELISA reader (Sunrise). The IC_50_ value was calculated by the GraphPad Prism programme.

### Zebrafish embryo-toxicity test

Zebrafish (Danio rerio) were maintained at 28°C under continuous flow of air and with automatic control of a 14-hour light/ 10-hour dark cycle. All zebrafish experiments were conducted under the approval of the institutional animal care and use committee (IACUC). Zebrafish embryo-toxicity test was performed according to guidelines of the Organization for Economic Co-operation and Development. The day prior to fertilization, male and female fish were left in mating tanks overnight. The next morning, the embryos were collected and transferred to a dish with E3 solution (5mM NaCl, 0.17mM KCl, 0.33mM CaCl_2_ and 0.33mM MgSO_4_, pH 7.0) and incubated at 28°C for 6 hours. Thirty-five embryos were produced and each of the 35 embryos were incubated and exposed to the range of concentrations between 12.5 μM and 1600 μM of TG1 for 1 day and 2 days. The number of dead embryos/fish and normal fish were recorded.

#### Assessment of drug combination synergism and combination index

In 1984, Chou and Talalay published a concept collaboratively addressing the term “Combination Index (CI),” explaining that the value quantitatively symbolized and analyzed drug potencies [21, 22]. Since the term was introduced, CI has been extensively used in scientific articles internationally. CI more than 1 means antagonism of the drugs; CI equal to 1 points to additive effects of the drugs; CI less than 1 implies synergistic effect of the drugs.

The mathematical formula for CI is as follows:

$$d_{1}=\left\{\frac{a}{a+b}\right\}{ED}_{50c}$$

$$d_{2}=\left\{\frac{a}{a+b}\right\}{ED}_{50c}$$

$$CI=\frac{d_{1}}{{ED}_{50(1)}}+\frac{d_{2}}{{ED}_{50(2)}}=\left\{\frac{a}{(a+b){ED}_{50(1)}}+\frac{b}{(a+b){ED}_{50(2)}}\right\}{ED}_{50c}$$

First, “d1” and “d2” signify doses of the drugs individually; second, “a” and “b” represent the drug ratios, respectively; third, “ED_50(1)_” and “ED_50(2)_” indicate the median effect doses of the drugs, respectively, while “ED_50c_” expresses the median effect dose of the drugs combined.

The principle and formula of CI can be applied to analysis and discussion of multiple drug potencies for cancer treatment. Therefore, this study aimed to calculate CIs to evaluate whether anticancer drugs and chemosensitizers exhibited synergistic effects or not.

#### Fluorescence examination of doxorubicin cellular uptake

Fluorescence images are used to analyze and investigate the effects of the multidrug resistance drug efflux pump in vitro. Because doxorubicin has a fluorescence signal, this property was used to detect and observe the images.

The breast cancer cells, MCF-7 and the multidrug resistance breast cancer cells, MCF-7/Adr were first placed in 48-well plates (Nunc) at a density of 2x10^4^ cells/well. After incubation of cells in the 48-well plates, each well was placed into different drug conditions (DOX, DOX+TG1 = 1:5) for 24 hours. Subsequently, the medium was removed and cells were rinsed 3 times with PBS. To fix the cells, 200μl 4% paraformaldehyde was added to each well for 15~20 minutes at room temperature. Then, the solution was removed and washed with PBS 3 times (4 minutes each). Finally, an excitation wavelength at 488 nm and emission wavelength at 520 nm were detected by inverted fluorescence microscope (Leica).

### Drug resistance associated proteins expression analysis

In order to investigate the multidrug resistance pathways between the anticancer drugs and the chemosensitizers, analysis of specific protein expression was conducted using western blotting.

Briefly, MCF7 or MCF7/Adr cells of the control group and drug of the experimental group (DOX, TG1 or DOX+TG1) were incubated for 72 hours. The cells were harvested with lysis solution (cell lysis buffer: Phosphatase Inhibitor Mix II: Protease Inhibitor Cocktail = 100:1:1) for 30 minutes at 4°C. The total protein levels were determined with a BCA assay (Bio-RAD, CA, USA) depends on the Bradford method. Equal amounts of protein (20 μg) were separated with 8%-15% SDS polyacrylamide gel electrophoresis and transferred to nitrocellulose membranes. The membrane was blocked with 5% BSA in PBST for 1 hour. Then, the primary antibodies included P-gp (1:500, mAb; Merck, MA, USA), MRP1(1:500, mAb; Merck, MA, USA), BCRP (1:500, mAb; Merck, MA, USA) or β-actin (1:1000, polyclonal Ab; Cambridge, UK) were incubated in 1% BSA dilution at 4°C overnight. On the next day, the membrane was washed for 1 hour with PBST. Then, HRP-conjugated secondary antibody solution in PBST dilution (1:1000) was incubated for 2 hours at room temperature.

To detect chemiluminescent signal, chemiluminescent substrate of 1:1(v/v) was added to the membrane. It was captured by BioSpectrum® 810 Imaging SystemTM (UVP). For image analysis, the band intensity of the target proteins was gained by software (N = 3 for each group).

### Statistical analysis

Data are reported as mean and standard deviation. The significance of the data was analyzed by Mann-Whitney U test between the control group and the experimental groups. Statistical testing was performed using GraphPad Prism (GraphPad Software, Inc., San Diego, CA), and a p-value < 0.01 was considered statistically significant.

## Results

### TG1 compound structure

**Fig 1:**
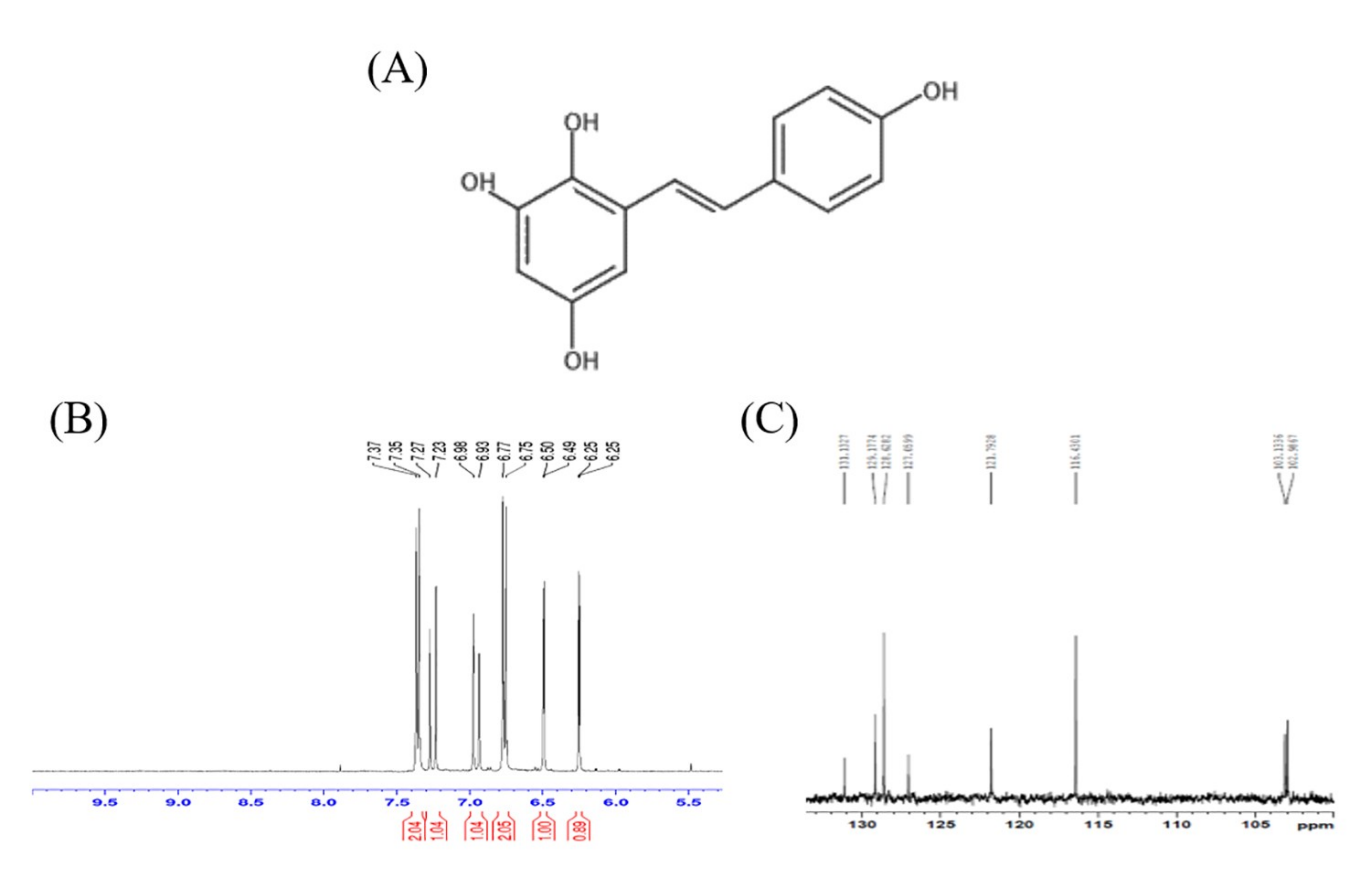
(A) Structure of TG1. (B) 1H NMR analysis of TG1. (C) 13C NMR analysis of TG1.

The compound structure of TG1 is shown in Fig 1, and was identified by NMR. TG1 ^1^H NMR (200 MHz, MeOD) δ 7.31, 6.72 (dt, J = 8.5, 2.9, 1.8 Hz; J = 8.7, 2.8, 2.0 Hz 2H; 2H, H-2’, H-6’, H-3’, H-5’), 7.21, 6.90 (d, J = 16.5 Hz; J = 16.4 Hz, 1H; 1H, H-7, H-8), 6.45, 6.20 ppm (d, J = 2.7 Hz; J = 2.7 Hz, 1H; 1H, H-3, H-5); ^13^C NMR (50 MHz, MeOD) δ 157.1, 150.3, 146.5, 136.4(C2, C4, C6, C4’), 130.1, 126.0(C1, C1’), 128.1, 120.7(C7, C8), 127.7, 115.4(C2’, C3’, C4’, C5’), 102.1, 101.9 ppm (C3, C5).

### Toxicity of TG1

**Fig 2:**
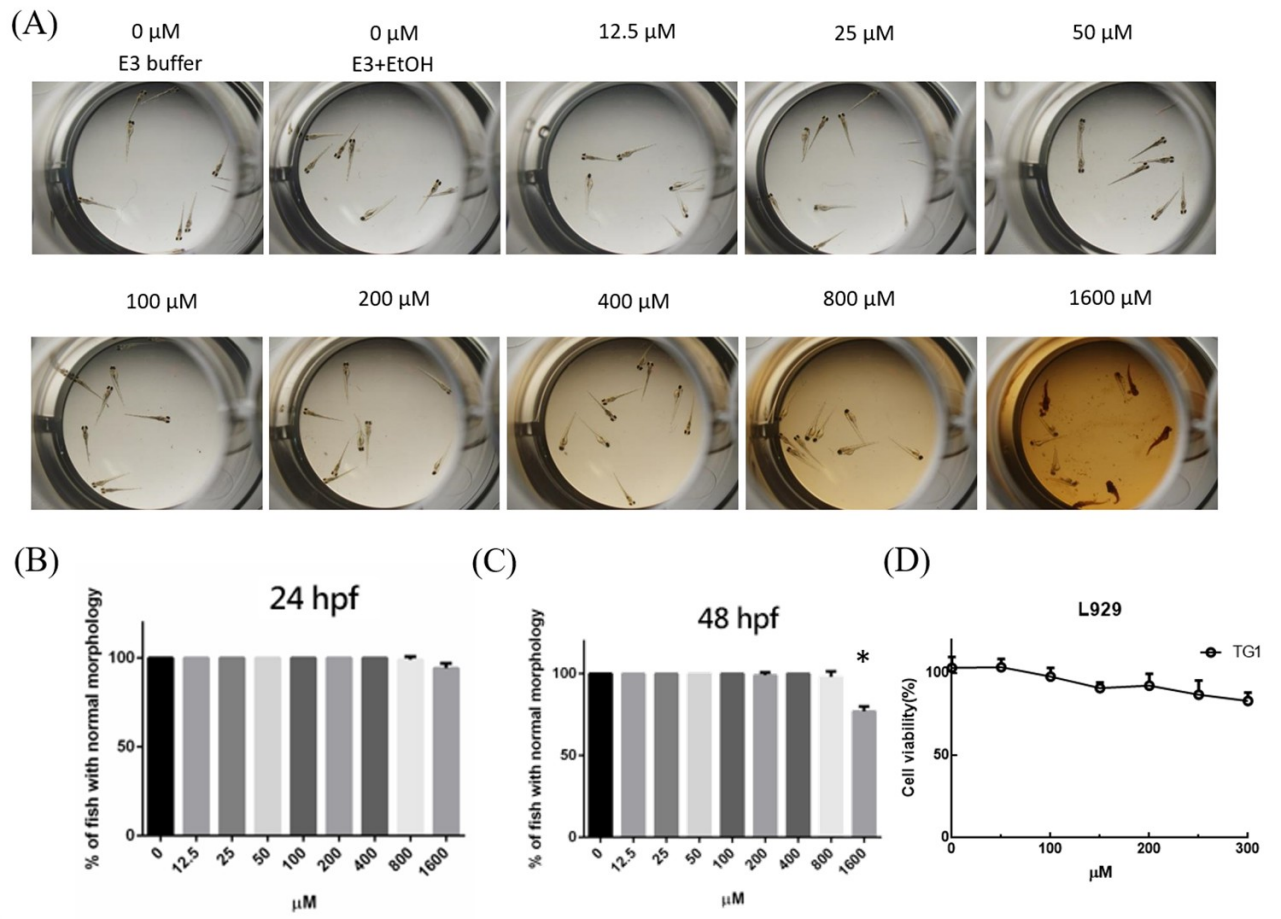
The effects of TG1 on the development of zebrafish embryos. (A) Representative images of zebrafish embryos exposed to TG1 at 48h. Survival rate after exposure to 12.5uM to 1600uM TG1 at (B) 24 and (C) 48 hpf. (D) Inhibition of TG1 on cell viability of L929 cells. (E3: E3 medium, for zebrafish embryos; EtOH: ethanol; hpf: hours post fertilisation) Data were shown by mean ± SD (standard deviation), n = 3. *p< 0.01, which indicated the groups had significant difference as comparing to control group.

The zebrafish animal system is thought to be a cost-effective tool for anticancer drug development [23, 24]. To evaluate the primary *in-vivo* toxicity of TG1, zebrafish embryos were exposed to various drug concentration of TG1 (range of concentrations between 12.5 μM and 1600 μM) at time points 24 and 48 hours post fertilization (hpf) (Fig 2A–2C). The survival rates were not significantly affected under 1600 μM of TG1 at 24 hpf. We found that zebrafish embryos exhibited a significantly decreased survival rate only when they were exposed to high concentration of TG1 (1600 μM) at 48 hpf.

L929 human fibroblast cells were used to analyze the cytotoxicity of TG1 *invitro*. Even if the concentration of TG1 was over 100 μM (Fig 2D), the L929 cells still maintained high cell viability, indicating the low toxicity of TG1 for fibroblast cells.

These results suggest no obvious in-vitro cellular toxicity or in-vivo developmental toxicity for TG1, suggesting its potential for further clinical applications.

### In-vitro antitumor effect of TG1

**Fig 3:**
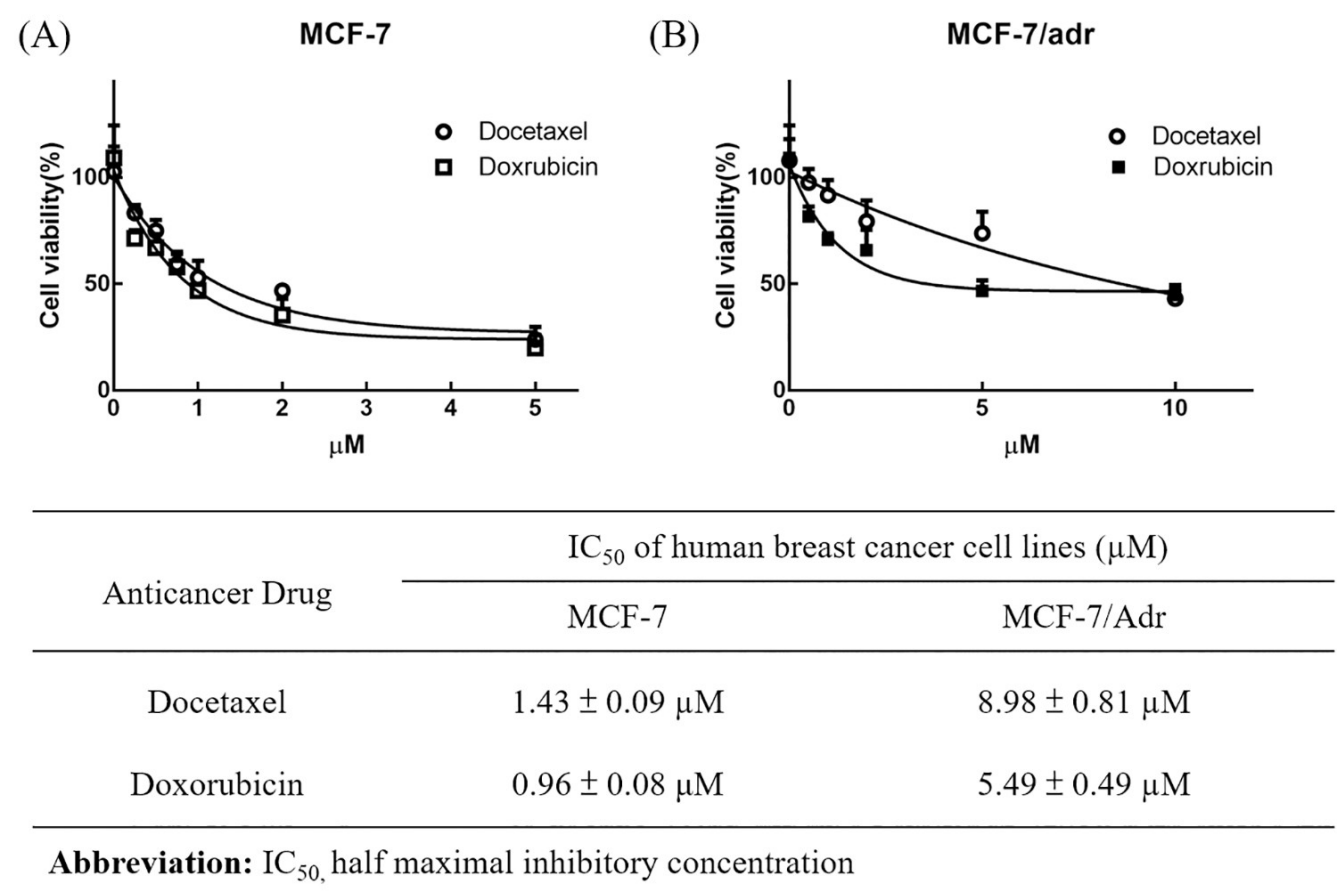
Inhibition of DOX or DTX on proliferation of (A) MCF-7 and (B) MCF-7/Adr cells. The IC_50_ values of DOX or DTX were calculated and showed on table. Data were shown by mean ± SD, n = 3.

**Fig 4:**
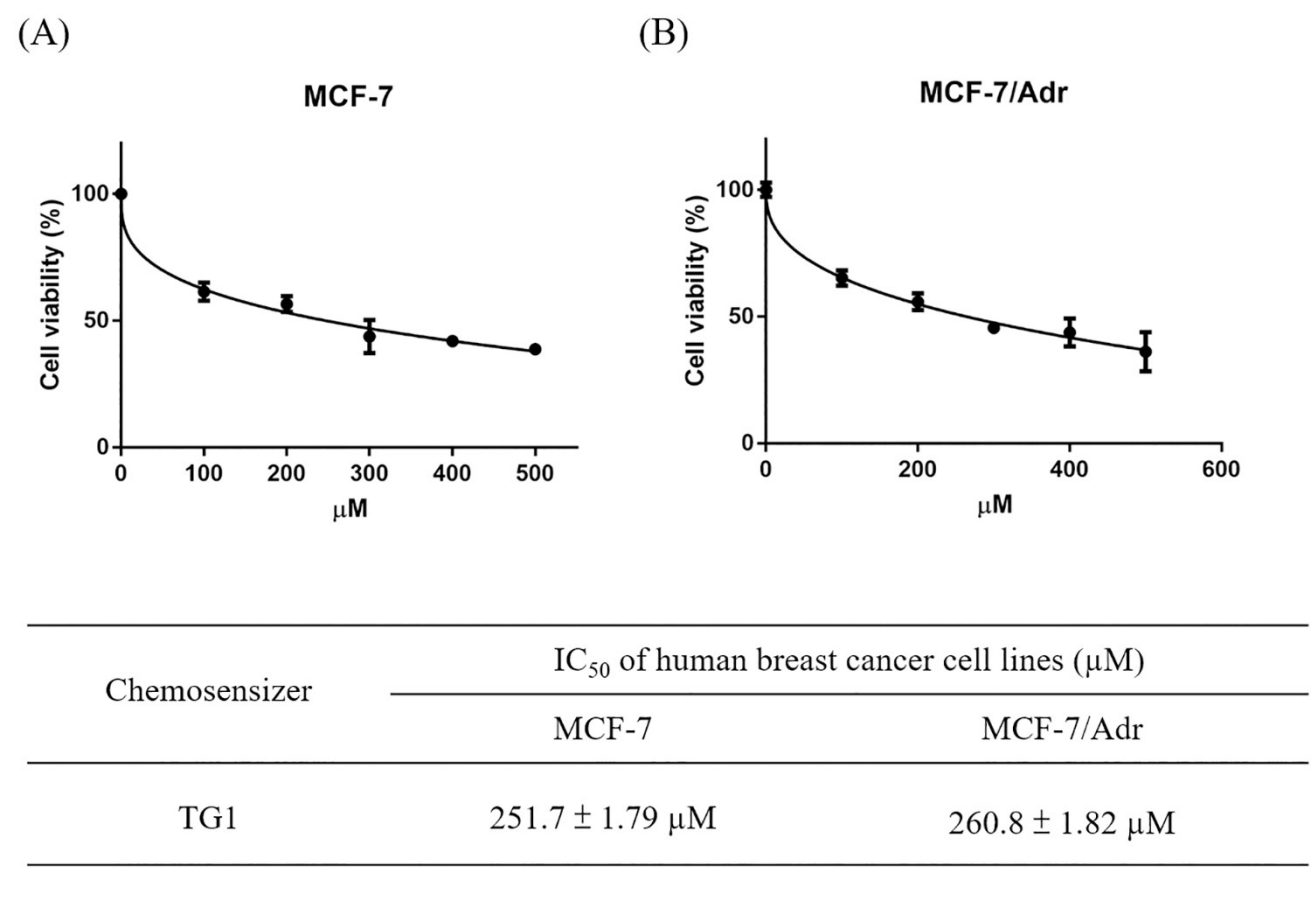
Inhibition of TG1 on proliferation of (A) MCF-7 and (B) MCF-7/Adr cells. The IC_50_ values of TG1 were calculated and showed on table. Data were shown by mean ± SD, n = 3.

For the breast cancer cells viability test, MCF-7 cells were examined for TG1 cytotoxicity and that of clinical drugs, docetaxel and doxorubicin. IC_50_ values of DTX and DOX were 1.43 ± 0.09 μM and 0.96 ± 0.08 μM, respectively (Fig 3A), while IC_50_ values of TG1 were 257.1 ± 1.79 μM (Fig 4A), indicating slight influence on the growth of breast cancer cells. MCF-7/Adr cells and human breast cancer MDR cells were also examined for cellular cytotoxicity of DTX, DOX and TG1. IC_50_ values of DTX and DOX were 8.98 ± 0.81 μM and 5.49 ± 0.49 μM, respectively (Fig 3B), which were higher than the IC_50_ values of MCF-7, indicating drug resistance effects of the breast cancer cells. IC_50_ of TG1 treated with MCF-7/Adr was 260.8 ± 1.79 μM (Fig 4B), which was similar to TG1 on MCF-7.

### Combination effect of TG1 and clinical drugs

**Fig 5:**
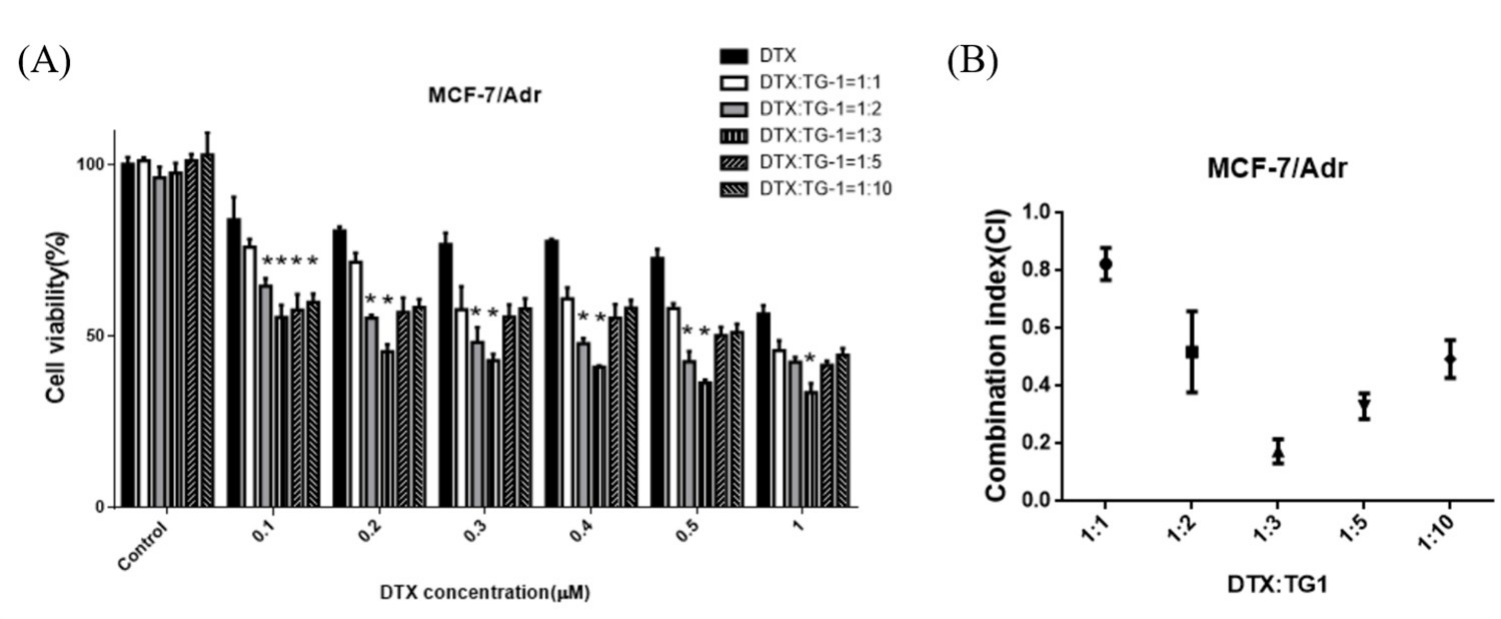
Combined effects of TG1 and DTX on inhibiting cell proliferation of MCF-7/Adr for 24 hours. (A)The cells were treated to different concentrations of DTX or combined with the chemosensitizer, TG1, in molar ratios of 1:1, 1:2, 1:3, 1:4 1:5 and 1:10. (B) Combination index of DTX and TG1 on different molar ratios for treating MCF-7/Adr cells. Data are shown as mean ± SD, n = 5. *p< 0.01, indicating significant differences between experimental and control groups.

**Fig 6:**
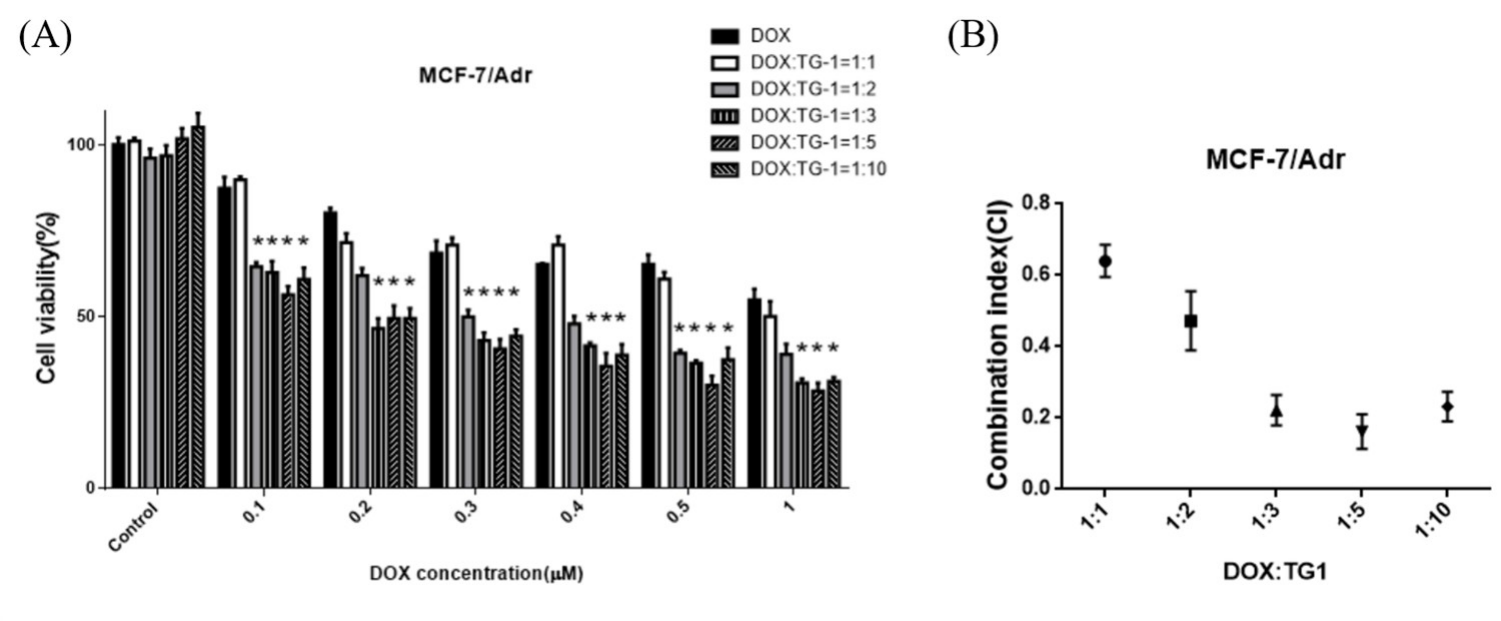
Combined effects of TG1 and DOX on inhibiting cell proliferation of MCF-7/Adr for 24 hours. (A)The cells were treated to different concentrations of DOX or combined with the chemosensitizer, TG1, in molar ratios of 1:1, 1:2, 1:3, 1:4 1:5 and 1:10. (B) Combination index of DOX and TG1 on different molar ratio for treating MCF-7/Adr cells. Data are shown by mean ± SD, n = 5. *p< 0.01, indicating significant differences between experimental and control groups.

For evaluation of cell cytotoxicity efficiency of anticancer drugs combined with TG1 for drug-resistant MCF-7/Adr cells, MTT assay was tested using the molar ratio of 1:1, 1:2, 1:3, 1:5 and 1:10 (DTX or DOX: TG1) (Figs 5A and 6A). TG1 was shown to effectively enhance the anti-cancer ability regardless of the molar ratio. Based on cell viability results, the combination index (CI) was calculated to ascertain the drug potencies of clinical drugs combined with TG1. As the CI value was less than 1, the drug combination exhibited synergistic effects (Fig 5B). The CI value of 1:1 (DTX: TG1) was 0.86, indicating weak synergistic effects. However, strong synergistic effects were shown with the molar ratio 1:3, and the CI value was lower than 0.2. For DOX and TG1 combination treatment, molar ratio 1:5 had strong synergistic effects and the CI value was 0.18 (Fig 6B). Thus, the 1:5 molar ratio of drugs and TG1 combination appears to be the most effective formulation against drug resistant breast cancer cells, suggesting their potential for further clinical application.

### In vitro cellular uptake for breast cancer cells

**Fig 7:**
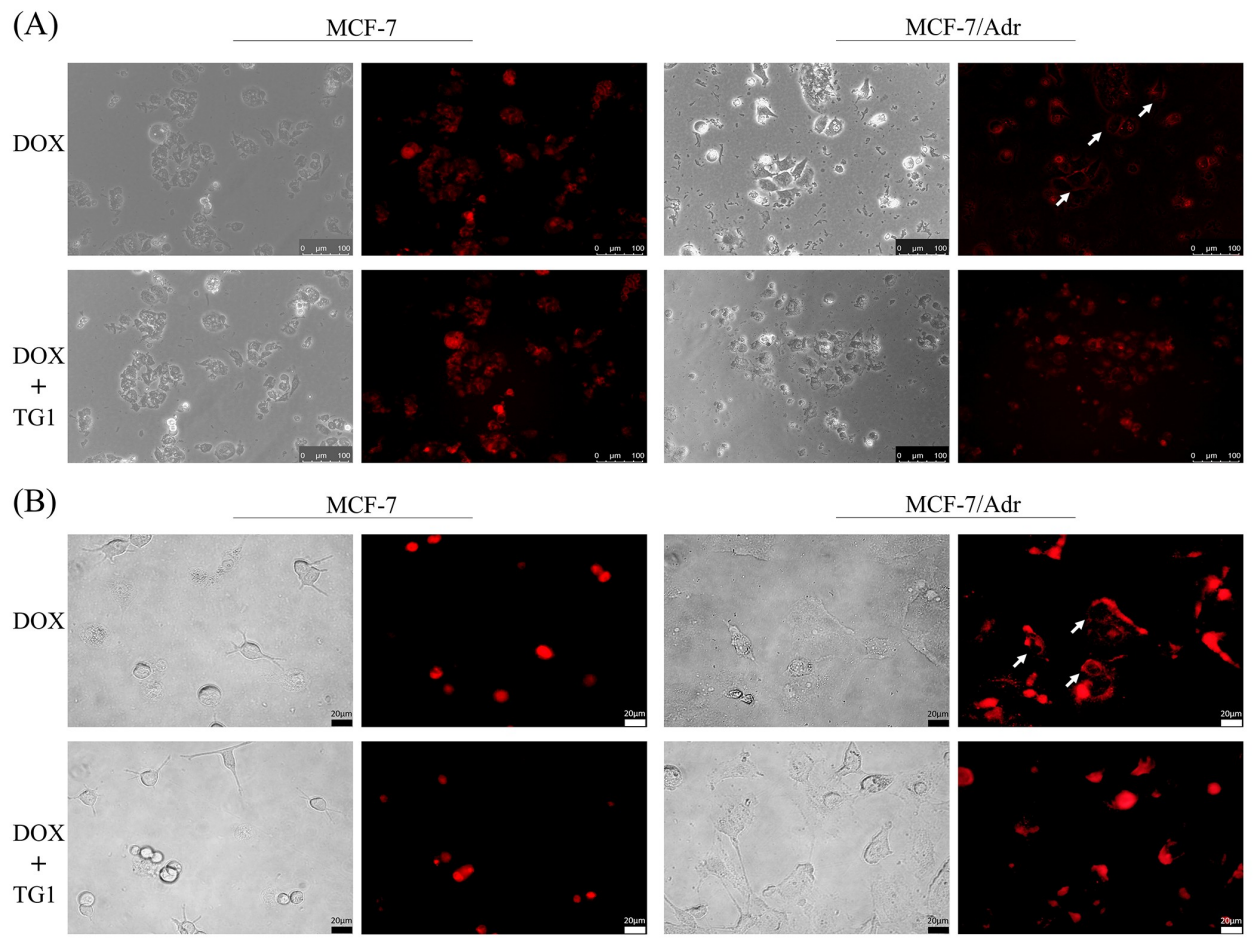
Fluorescence images of cellular uptake of DOX or DOX combined with TG1 in MCF-7 and MCF-7/Adr by inverted fluorescence microscope. (A) 20x images (B) 40x images.

To visualize MDR effects, the fluorescence signal of DOX was evaluated to detect cellular uptake of breast cancer cells (Fig 7). In MCF-7 cells, both DOX treatment and DOX/TG1 treatment were found in breast cancer cells, however, distinct images were seen in MCF-7/Adr. MCF-7/Adr images showed that the fluorescence signal of the DOX was found in the outer part of nucleus and cytoplasm because of the action of the drug efflux pump; however, the DOX fluorescence signal of DOX/TG1 treatment was detected in the cell nucleus, indicating good internalization by TG1.

### Presentation of MDR related transporters

**Fig 8:**
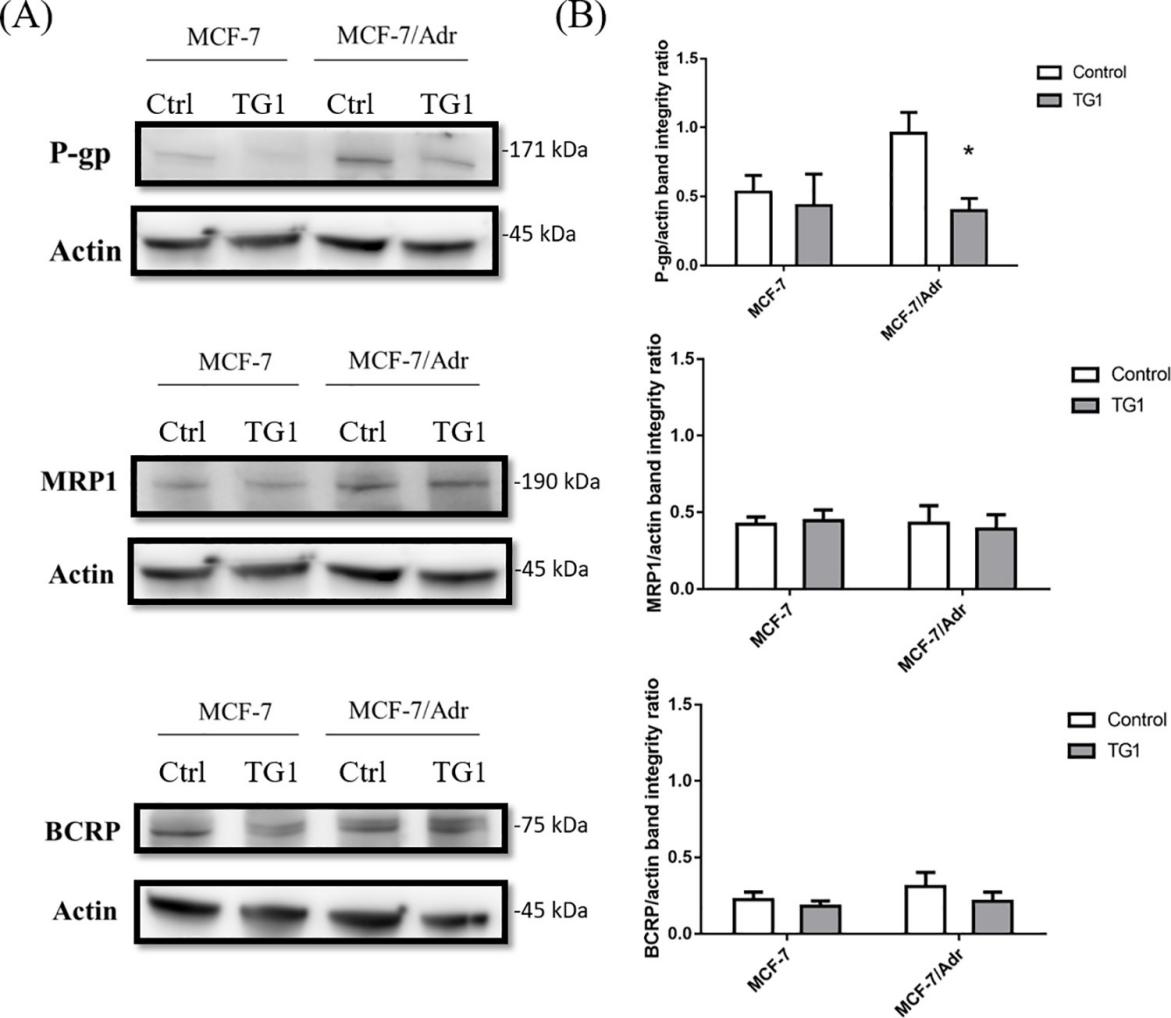
Effect of TG1 on expression of MDR related transporter in MCF or MCF-7/Adr cells. (A) Expression of P-gp, MRP1 and BCRP using western blot on MCF-7 or MCF-7/Adr cells. (B) Quantitative data are shown as mean ± SD, n = 3. *p< 0.01, which indicates significant differences between the experimental and control groups.

Western blotting was used to assess multidrug resistance by ABC transporters, by analyzing the expression of different drug efflux pumps. Comparisons of the expression of P-gp, MRP1, and BCRP in MCF-7 and MCF-7/Adr are shown in Fig 8. P-gp expression in MCF-7/Adr was much higher than MCF-7, but the expression of MRP1 and BCRP in MCF-7/Adr was similar to MCF-7. However, when MCF-7 and MCF-7/Adr were treated with TG1 for 72 hours, P-gp expressions were also suppressed in both (Fig 8), with highly intense P-gp inhibitory effects in MCF-7/Adr. Nevertheless, neither MRP1 nor BCRP expression was affected after TG1 treatment.

**Fig 9:**
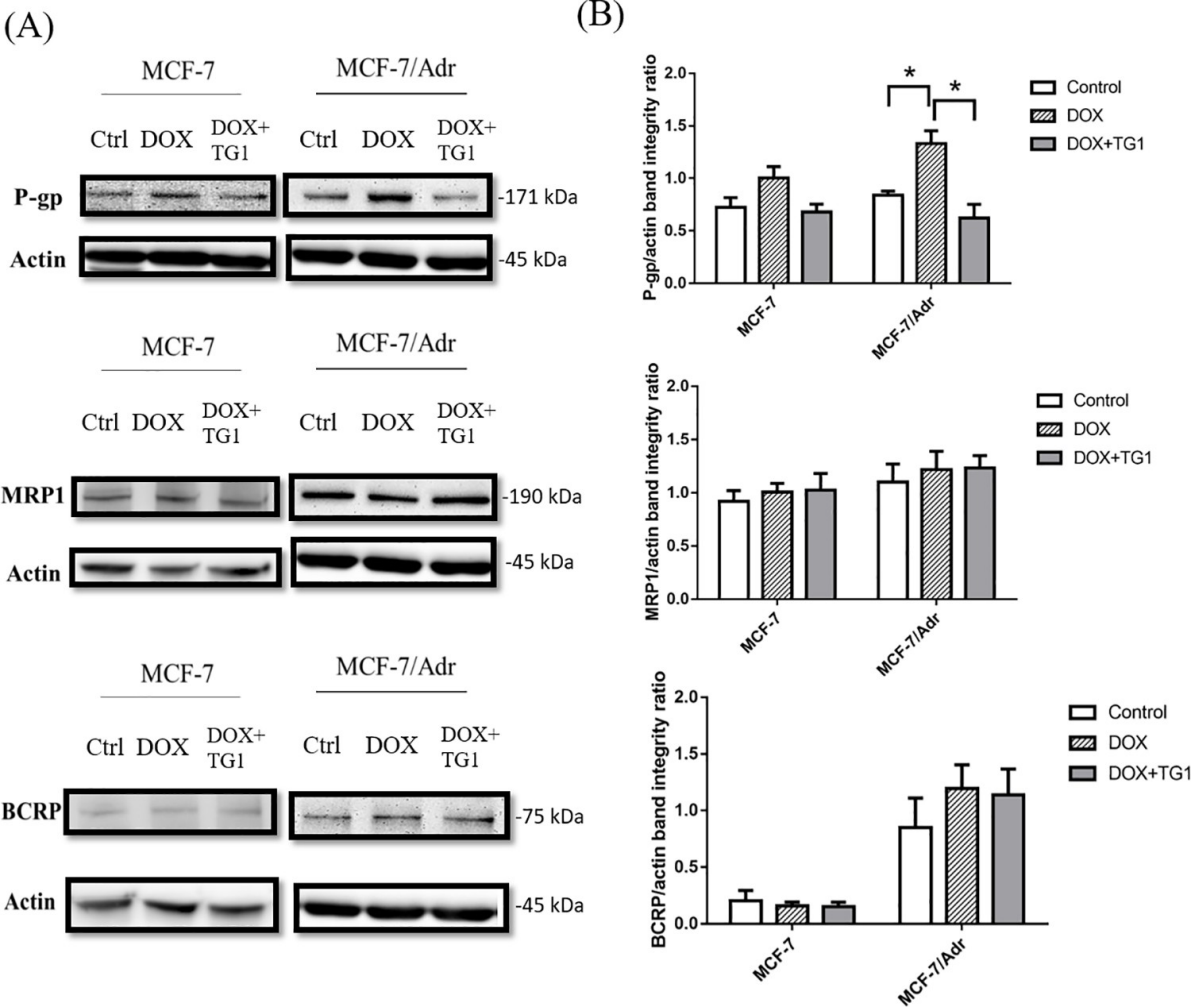
Effects of DOX combined with TG1 on expression of MDR related transporter in MCF-7 or MCF-7/Adr cells. (A) Expression of P-gp, MRP1 and BCRP using western blot on MCF-7 or MCF-7/Adr cells. (B) Quantitative data are shown as mean ± SD, n = 3. *p< 0.01, which indicates significant differences between experimental and control groups.

To evaluate the chemosensitizing effects of TG1, cancer drugs were combined for treatment. After DOX treatment in MCF-7/Adr, P-gp expression was higher than the groups without any drug treatment. However, no increases were shown in MRP1 and BCRP expression after the treatment. Next, when DOX and TG1 were treated together in MCF-7 and MCF-7/Adr, P-gp expression was suppressed in both, especially in MCF-7/Adr (Fig 9). These results indicate that TG1 may play an important role in inhibiting the P-gp drug efflux pump expression, which prevents anticancer drugs from being pumped out to the extracellular region.

## Discussion

The present study attempted to find a safe and effective way to solve chemotherapy failure due to severe side effects of anticancer drugs and the difficulty of sustaining clinical treatment. In particular, chemotherapy drugs have limited efficacy in breast cancer due to multidrug resistance generated by cancer cells against anticancer drugs. In this study, the novel compound, TG1, showed low toxicity and potential application for breast cancer patients, especially those with multidrug resistance.

Many scientists have tried various means to ameliorate chemotherapy effects, including the use of non-toxic drug carriers or using new herbal compounds [25, 26]. In the present study, the properties of cytotoxicity, combination index, and drug efflux pump expression of TG1 were investigated. TG1 was derived from THSG deglucosylation, and it has a chemical structure with multiple hydroxyl groups. Previous study indicated that hydrogen bonds were formed between the hydroxyl groups of inhibitors and the R site of drug efflux pump, P-gp [27]. Thus, the modification of THSG may have improved the reduction of drug efflux effect via P-gp.

Zebrafish are a model vertebrate that has been useful in human drug screening because humans and zebrafish have many of the same orthologous genes [28], and its advantages as a material for drug development are rapid development, high fecundity and relatively inexpensive [29]. Therefore, it is a beneficial model for drug safety assessment. Previous studies have revealed that the drug of the atractylodin induced the mortality with the LC_50_ 52 μM [30]. However, zebrafish embryos exposed to TG1 under concentration of 800 μM showed a great in-vivo survival rate.

Based on results of previous studies, resveratrol acted as a fine chemosensitizer to support the anti-cancer efficacy when combined with clinical drugs, DOX or DTX [31–33]. The authors pointed out that the combination of resveratrol with DTX or DOX in the molar ratio of 1:15000 or 1:1 to 1:6 produced moderate synergistic effects (CI< 0.7~0.85) and slightly antagonistic (CI> 1), respectively. Xiong Guo *et al*. also showed the CI of DTX combined with resveratrol in the ratio of 2:1, 1:1, and 1:2 was 0.92, 0.64, and 1.2, which indicated that in the ratio of 1:2 had an antagonistic effect [25]. In the present study, the combination indexes had better synergistic effects than resveratrol. The combination treatment of anticancer drugs and TG1 may have the highest effectiveness in the molar ratio of 1:3 (DTX: TG1, CI = 0.19) and 1:5 (DOX: TG1, CI = 0.18).

One of the important pathways was the drug efflux pumps located on the cell membrane, called ABC transporters [34]. Among these multiple transporters, P-gp was shown to play a vital role in developing resistance to anticancer drugs by overexpression in breast cancer cells [35–37]. Therefore, in the present study, two cell lines were used to compare the effects; MCF-7, and MDR cancer cells, MCF-7/Adr, which was a P-gp overexpressing derivative cells [38]. A better synergistic effect was shown in the present study than resveratrol according to the combination indexes. The combination treatment of anticancer drugs-DOX and TG1 appear to have the most effectiveness in the molar ratio of 1:5, indicating that the anticancer drugs can be reduced and maintained their effects at the same time.

In addition, TG1 also inhibits P-gp expression when the anticancer drugs were reacted against cancer cells, which demonstrated better effects than resveratrol [39]. Therefore, it would result in assisting anticancer drugs to exert the cytotoxicity via downregulating the drug efflux pump expression, especially performed on multidrug resistance cancer cells. Xue et al. determined metformin was an inhibitor rather than substrate of P-gp by bidirectional transport assay in MDR1-MDCK cells [40]. Resveratrol had a similar bi directional transport result with metformin [41]. Furthermore, many researches were investigated resveratrol as a P-gp inhibitor on cancer cells overexpressing P-gp [42–44]. Since TG1 has similar chemical structure with resveratrol, and the potential P-gp inhibitor could be predicted by structure [27]. TG1 might be a P-gp inhibitor to modulate DTX/DOX resistance effects, but it needs more investigation in the future.

## Conclusion

The novel derivative TG1 exhibits no obvious in-vitro cellular toxicity or in-vivo developmental toxicity. The combination of anticancer drugs and TG1 promotes cytotoxicity against breast cancer cells because of its inhibitory effects on multidrug resistance, especially for p-glycoprotein drug efflux pump suppression. Therefore, this novel combinational therapy exhibits potential synergistic effects on treating breast cancer cells, especially those with multidrug resistance.

## Supporting information

S1 Raw images (PDF)
