# Supplementary material for: Reduction of breast tumor drug resistance by 2,3,5,4’-tetrahydroxystilbene for exhibition synergic chemotherapeutic effect
Source: PLoS One. 2021 Dec 7;16(12):e0260533. doi: 10.1371/journal.pone.0260533 (PMC8651109; doi:10.1371/journal.pone.0260533)
Supplement: S1 Raw images — (PDF) [file pone.0260533.s001.pdf]

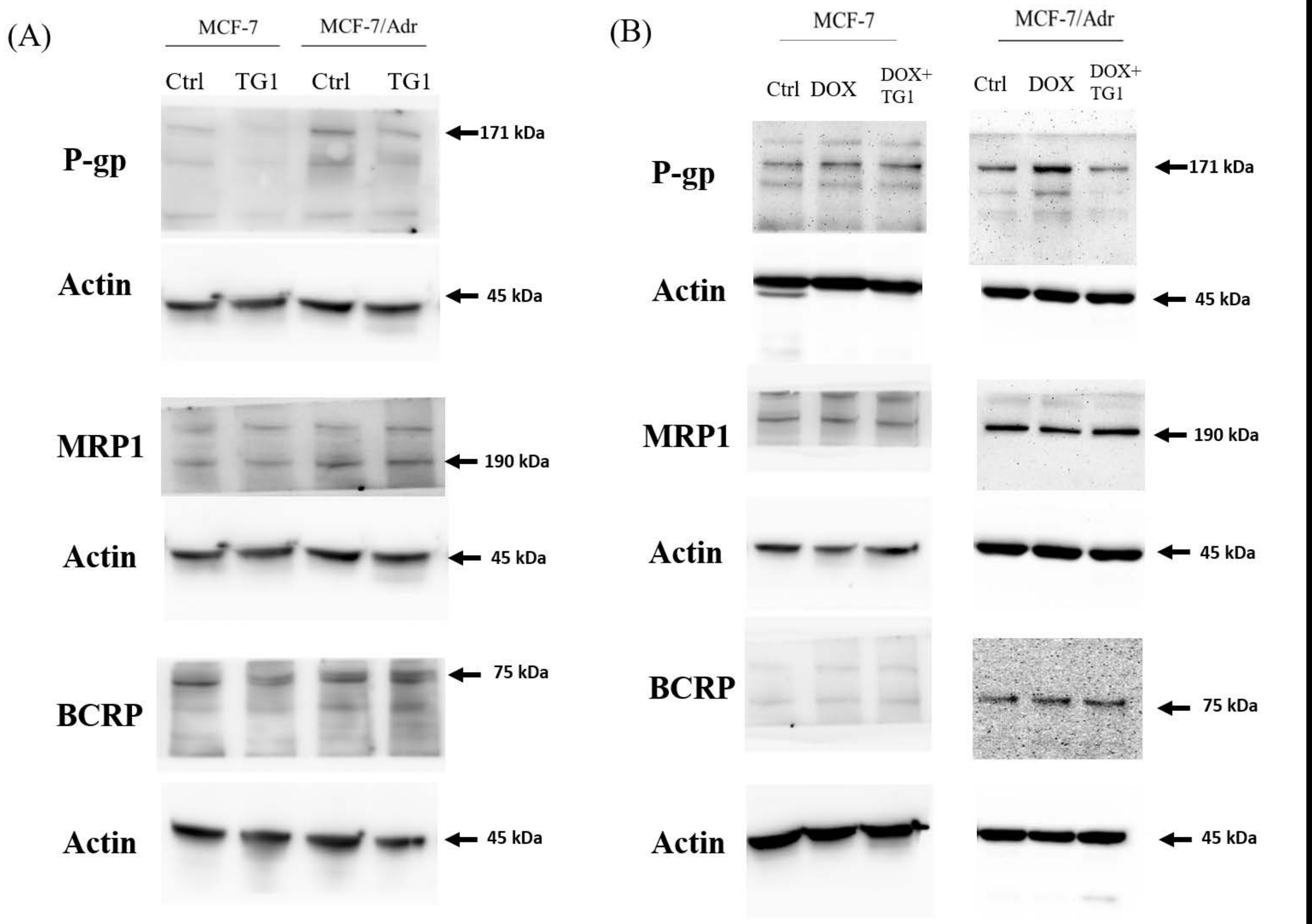

(A)
MCF-7
MCF-7/Adr
Ctrl
TG1
Ctrl
TG1
P-gp
171 kDa
Actin
45 kDa
MRP1
190 kDa
Actin
45 kDa
BCRP
75 kDa
Actin
45 kDa
(B)
MCF-7
MCF-7/Adr
Ctrl
DOX
DOX+ TG1
Ctrl
DOX
DOX+ TG1
P-gp
171 kDa
Actin
45 kDa
MRP1
190 kDa
Actin
45 kDa
BCRP
75 kDa
Actin
45 kDa

Supplemental Fig. S1 (A)Full scan blots from Figure 8A for P-gp, MRP1 and BCRP, (b) Full scan blots from Figure 9A for P-gp, MRP1 and BCRP.
